# The effectiveness of eye tracking in the diagnosis of cognitive disorders: A systematic review and meta-analysis

**DOI:** 10.1371/journal.pone.0254059

**Published:** 2021-07-12

**Authors:** Zicai Liu, Zhen Yang, Yueming Gu, Huiyu Liu, Pu Wang

**Affiliations:** 1 Department of Rehabilitation Medicine, Yue Bei People’s Hospital, Shaoguan, Guangdong, China; 2 Histology and Imaging platform, Core Facilities of West China Hospital, Sichuan University, China; 3 Rehabilitation College of Gannan Medical University, Ganzhou, Jiangxi, China; 4 Department of Rehabilitation Medicine, The 7th Affiliated Hospital of Sun Yat-Sen University (Shenzhen), Shenzhen, Guangdong, China; 5 Guangdong Engineering and Technology Research Center for Rehabilitation Medicine and Translation, Guangzhou, China; Mayo Clinic Minnesota, UNITED STATES

## Abstract

**Background:**

Eye tracking (ET) is a viable marker for the recognition of cognitive disorders. We assessed the accuracy and clinical value of ET for the diagnosis of cognitive disorders in patients.

**Methods:**

We searched the Medline, Embase, Web of Science, Cochrane Library, and Pubmed databases from inception to March 2, 2021, as well as the reference lists of identified primary studies. We included articles written in English that investigated ET for cognitive disorder patients—Mild cognitive impairment (MCI), Alzheimer’s disease (AD), Amyotrophic lateral sclerosis (ALS), and dementia. Two independent researchers extracted the data and the characteristics of each study; We calculated pooled sensitivities and specificities. A hierarchical summary of receiver performance characteristics (HSROC) model was used to test the diagnostic accuracy of ET for cognitive impairment (CI).

**Findings:**

11 studies met the inclusion criteria and were included in qualitative comprehensive analysis. Meta-analysis was performed on 9 trials using Neuropsychological Cognitive Testing (NCT) as the reference standard. The comprehensive sensitivity and specificity of ET for detecting cognitive disorders were 0.75 (95% CI 0.72–0.79) and 0.73 (95% CI 0.70 to 0.76), respectively. The combined positive likelihood ratio (LR+) was 2.74 (95%CI 2.32–3.24) and the negative likelihood ratio (LR−) was 0.27 (95%CI 0.18–0.42).

**Conclusions:**

This review showed that ET technology could be used to detect the decline in CI, clinical use of ET techniques in combination with other tools to assess CI can be encouraged.

## Introduction

Cognitive function includes learning and memory, language, visuospatial, executive, and psychomotor [1]. Cognitive impairment (CI) was considered as injury in two or more areas of cognition [2], prevalence of CI is as high as 35–50% [3]. The rising incidence of CI has become a serious health problem due to an aging population [4]. It has been regarded as a clinical state with characteristics similar to those of normal aging and mild dementia [5]. Research has shown that patients with CI have a high rate of missed diagnosis and delayed diagnosis [6].

Early diagnosis of CI can contribute to specific clinical classification and prognosis and progression of the disease, as well as to treatment. The mini-mental state examination (MMSE) and Montreal Cognitive Assessment (MOCA) were general screening tools for CI [7, 8], these tools have proven to be highly sensitive [9]. However, both age and education level affect MMSE scores [10]. Existing bedside tools MMSE and MOCA are not sensitive to milder impairment [11], results grading resolution is not high (absent/slight/moderate) [12], and iterative feedback based on a large database is also absent [13], the examiner who use them need to be trained to make the results more reliable [14]. Eye tracking (ET), by contrast, is a new technique that objectively measures eye movement and the location of a subject’s gaze [15], it is becoming increasingly popular because it can provide better quantitative parameters for big data analysis. And ET provides a susceptive, economical, and noninvasive marker for change or deterioration in cognition [16, 17]. At present, the evidence on ET mainly focuses on the diagnosis of CI in neurodegenerative diseases, and there is no consensus on whether it is more sensitive or specific than the existing cognitive assessment [18]. Although indicators of oculomotor nerve function have been shown to be related to cognition [19, 20], and ET can be used as a diagnostic biomarker to evaluate executive function [21], but the effectiveness of ET in the diagnosis of CI still has no moderately convincing evidence.

Therefore, we conducted a systematic review and meta-analysis of the use of ET techniques to diagnose CI. We undertook this systematic review to summarize the existing evidence and evaluate the diagnostic value of ET in CI.

## Methods

### Information sources

In March 2021, the following English databases were searched for eligible studies: Embase (via website), MEDLINE (via website), the Cochrane library (via website), PubMed (via website), and Web of Science (via website). We searched the databases from inception to March 2, 2021. We also searched references for each target study.

### Search strategy

Two authors (Zicai Liu and Zhen Yang) conducted the search strategies. The search entries we used were as follows: (“Cognitive Impairment” OR “cognitive functions” OR “cognition” OR “Cognitive dysfunction” OR “Cognitive decline” OR “cognitive disorders”) AND (“eye-tracking” OR “gaze-tracking” OR “eye movement” OR “oculomotor” OR “fixation tracking” OR “saccade” OR “eye task”) AND (“diagnose” OR “Diagnosis” OR "Sensitivity and Specificity" OR “diagnostic accuracy” OR “accuracy” OR “screening test” OR “sensitivity specificity”).

### Eligibility criteria

#### Type of study

Randomized control, case-control, and cohort studies, and other types of studies were included, the true or false positive or negative rates (i.e., TP, FP, FN, TN) can be obtained directly or indirectly from the original study [22]. The original investigation must use ET to measure ocular data. Only studies published in English were included, both animal experiments and systematic reviews, as well as conference reports and case reports, and so on, were excluded [23].

#### Patients

Our target condition of interest was CI, therefore, the study must include people with CI, there are no limitations for age, region, sex, and race.

#### Index tests

In the included studies, the index test was ET.

#### Outcomes

The primary indicators were the sensitivity and specificity of ET in the included studies.

#### Reference standards

Neuropsychological cognitive testing (NCT) was regarded as the reference standard in our research. NCT includes tests of executive function, language, visuospatial skills and memory and so on; a patient with a lower or higher test score than normal was considered NCT positive, if the score falls within the range of normal people, it was considered cognitively normal. therefore, it can be determined whether the patient has CI.

### Data extraction

Two investigators independently extracted and managed the data, including the first author’s last name and year of publication; sample size; ET tasks; sensitivity; specificity; prevalence; TP; FP; TN; FN. Then the data were aggregated, the dispute was resolved in consultation with the author (Pu wang).

### Quality evaluation

Two investigators independently used a Quality Assessment of Diagnostic Accuracy Studies (QUADAS-2) [24]. the risk of bias and applicability were analyzed using Review Manager 5.3 [25]. Deek’s Funnel Plot and Egger’s method were used to test the publication deviation.

### Data synthesis and statistical analysis

Review Manager 5.3 was used to calculate TN, FP, and FN according to the total sample size of sensitivity and specificity provided in the original paper. the forest maps were generated using the Meta-Disc software, which showed the comprehensive sensitivity and specificity, diagnostic odds ratio (DOR), LR+ and LR-, and its 95% confidence interval (CI). The pooled dates of the included studies were calculated by using a hierarchical summary ROC model (HSROC) [25]. The analyses were conducted in Stata 12.0 and Meta-disc software.

## Results

### Results of the search

**Fig 1:**
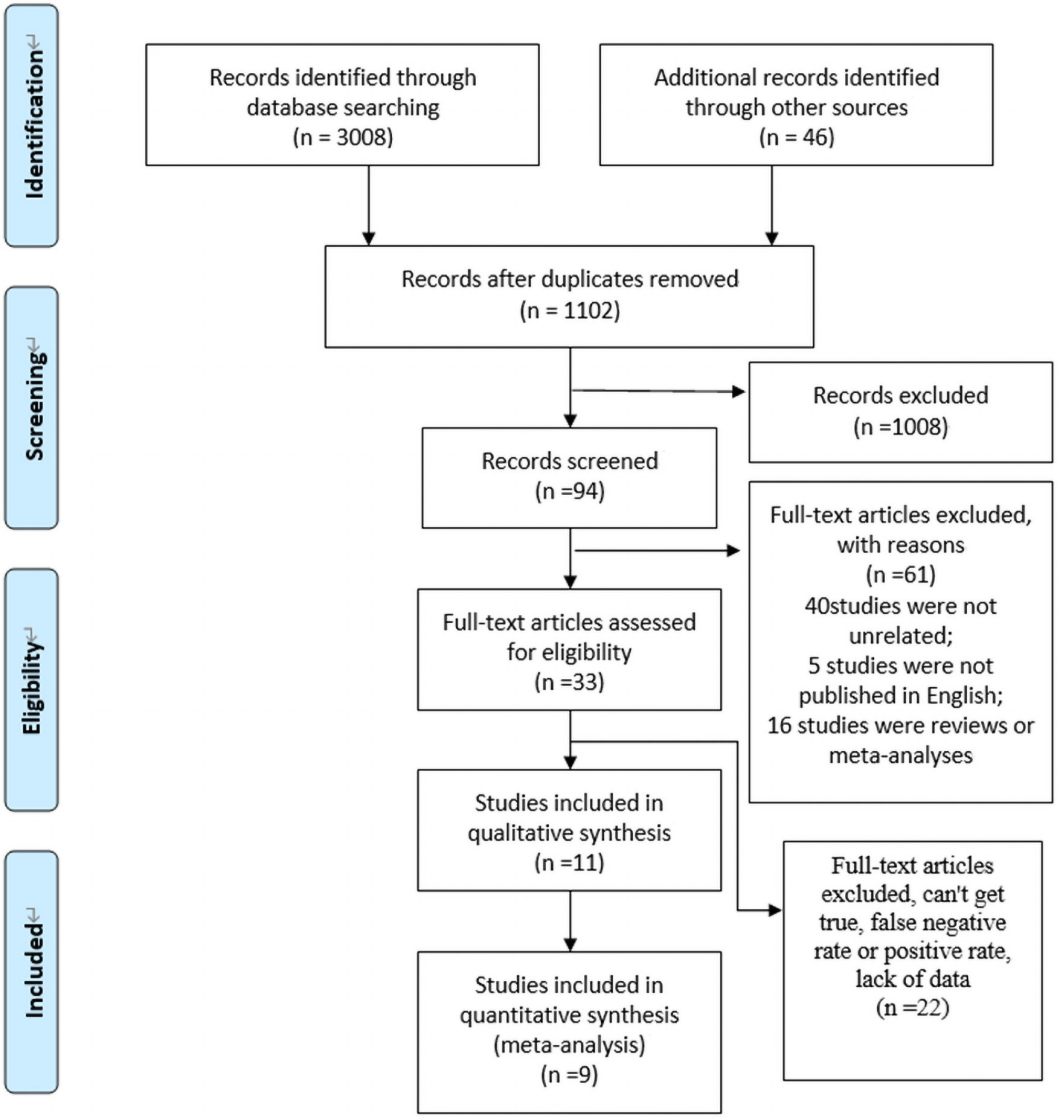
Retrieval process and selection process.

A total of 3054 literatures were retrieved from the five databases MEDLINE/ PubMed (n = 707), Embase (n = 1722), Cochrane (n = 145), Web of Science (n = 434) databases. We traced the list of references from the preliminary study and identified another 46 records, we excluded 1952 duplicate records. Then a total of 94 potentially related studies were identified by reading titles and abstracts to eliminate 1008 unrelated records. Of the 94 records, 40 studies were not diagnostic accuracy studies or unrelated to CI, 5 studies were published in non-English, and 16 studies were reviews or meta-analyses. In the end, the qualitative descriptive analysis included 11 studies [26–36], 9 comparative studies met all criteria and were included in a quantitative meta-analysis. Fig 1 shows our retrieval process and selection process.

### Characteristics of the studies

Table 1 lists the characteristics of the included studies. Diseases that cause CI included mild cognitive impairment (MCI), Alzheimer’s disease (AD), amyotrophic lateral sclerosis (ALS), dementia. the prevalence of CI ranged from 0.20 to 0.66, Sample sizes ranged from 42 to 522, it was published from 2011 to 2020. Regarding the description of the eye movement tracking, 3 studies used visual paired-comparison (VPC) [26–28], while most other studies used the regular eye gaze tracking task (such as Saccade, fixation task, and so on). We found that Poletti et al. used eye-tracking combined with common tasks of ET-based neuropsychological assessment [29], there are three eye movement parameters (including the 6-word test, RME test, and the MSCT test). Keller et al. used oculomotor testing (including the CPM test and the D2-Test) with two kinds of tasks [30]. One of the studies included patients with different types of dementia in dementia state, including AD and the behavioral variant of frontotemporal dementia (bvFTD), and other language-dominated dementias [36].

**Table 1:** The characteristics of the included studies.

Study (author year)	Sample size	Prevalence	participant	Eye tracking	Reference test	TP	FP	TN	FN	Sensitivity (95%CI)	Specificity (95%CI)
Haque 2019	296	0.38	MCI/AD	VisMET	NCT	97	45	136	17	0.85	0.75
Nie 2020	250	0.32	MCI	VPC	NCT	42	48	123	37	0.53	0.72
Pereira 2020	127	0.66	MCI/AD	Eye-tracking test	NCT	60	14	30	23	0.72	0.69
Lagun2011	60	0.50	MCI/AD	VPC	NCT	29	7	23	1	0.967	0.772
Jiang 2019	336	0.45	MCI	Visual tracking task	NCT	98	36	148	54	0.64	0.8
Gills 2020	55	0.20	MCI	VPC	NCT	10	15	29	1	0.9	0.65
Chehrehnegar 2019	120	0.50	a-MCI/AD	AST/PST gap task	NCT	36	23	36	4	0.9	0.61
Oyama 2019	80	0.66	MCI/dementia	Gaze task	NCT	44	7	20	9	0.8302	0.7407
Mengoudi2020	522	0.23	Dementia	Eye-tracking test	NCT	44.8	16.5	33.6	5.16	0.8967	0.67
Keller 2015	80	0.60	ALS	Oculomotor testing	NCT	21	3	29	27	0.44(CPM)	0.92
						18	3	29	30	0.38(D2-Test)	0.92
Poletti 2017	42	0.50	ALS	Visual tracking task	NCT	17	7	14	4	0.80(6-word)	0.667
						15	6	15	6	0.737(MSCT)	0.714
						14	6	15	7	0.684(RME)	0.737

"Table 1: Study characteristics.

MCI = Mild cognitive impairment, AD = Alzheimer’s disease, ALS = Amyotrophic lateral sclerosis, VisMET = Visuospatial Memory ET Task; PD = Parkinson’s disease; TP = true positive; FP = false positive; TN = true negative; FN = false negative; VPC = visual paired-comparison; NCT = Neuropsychological cognitive testing; AST = anti-saccade trials; PST = pro-saccade trials; CPM = Raven’s coloured progressive matrices; MSCT = Modified Card Sorting Test; RME = Reading the Mind in the eyes test".

### Methodological quality

**Fig 2:**
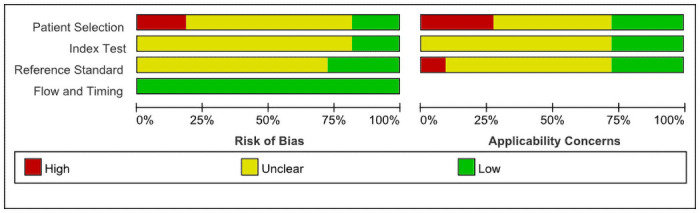
Risk of bias and applicability concerns.

**Fig 3:**
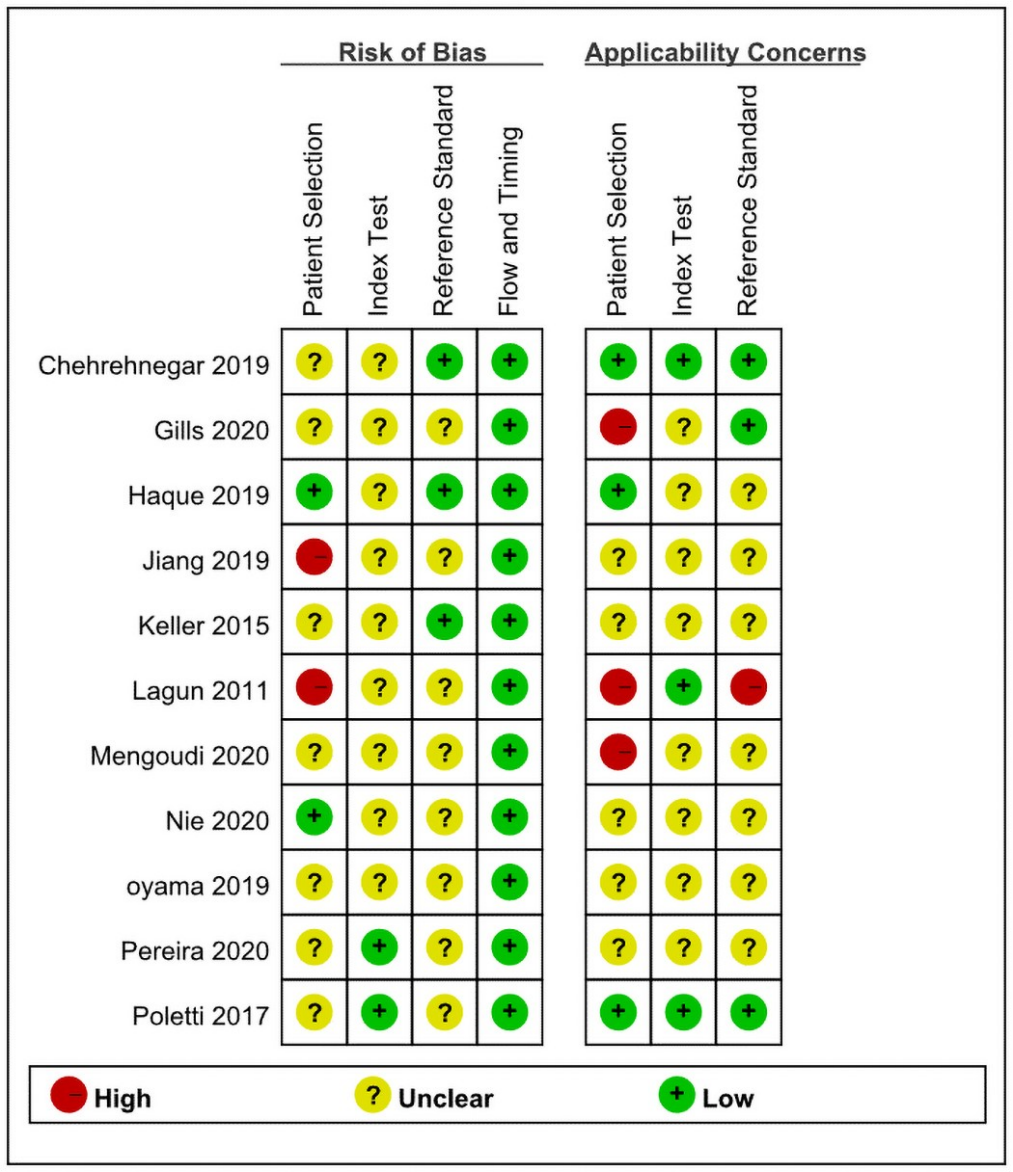
Risk of bias and applicability concerns.

Our assessment of the risk of bias and applicability for each area of the included studies can be seen in Figs 2 and 3. and the risk of bias was large because of patient selection, the Flowing and Timing component was low risk.

### Meta-analysis

**Fig 4:**
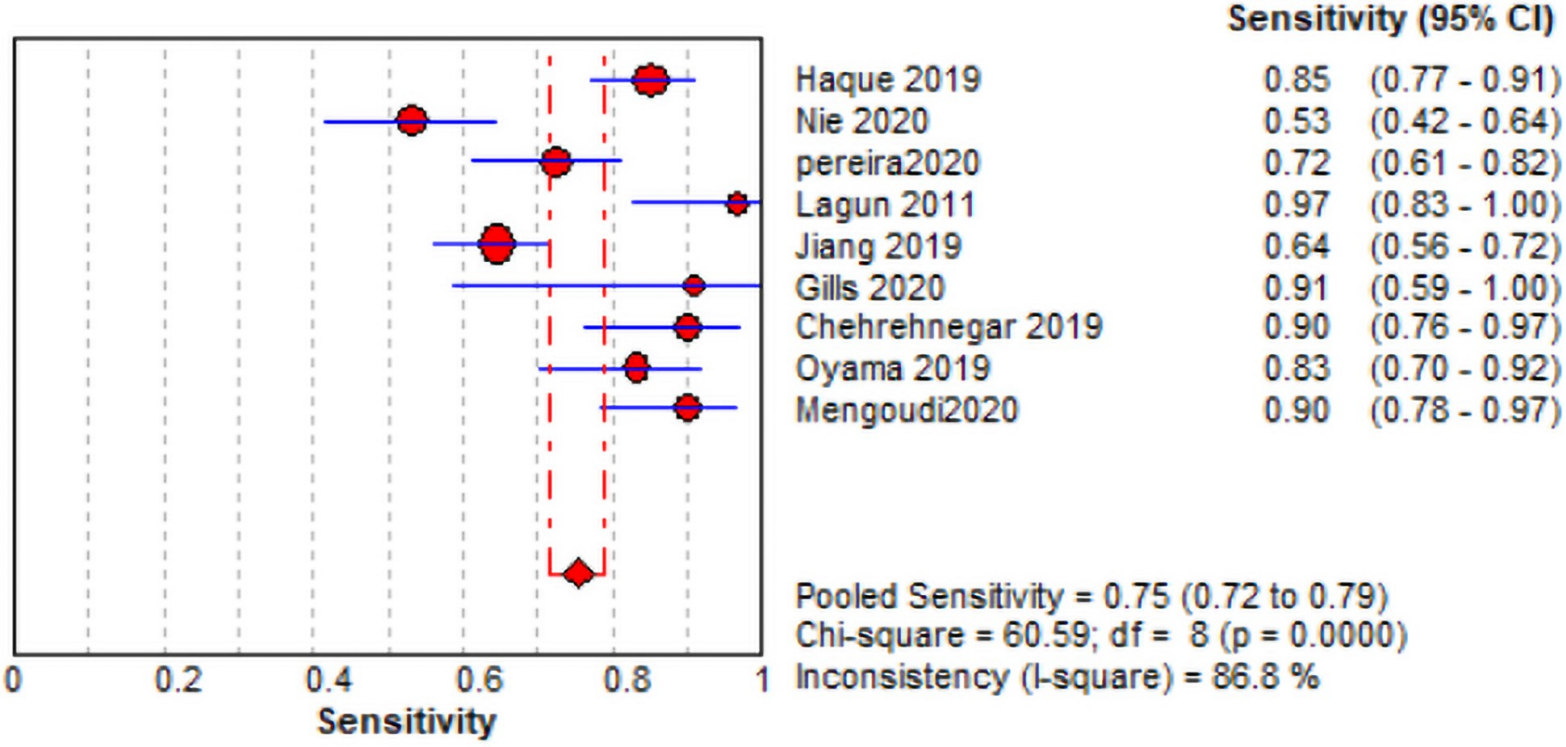
Forest sensitivity map (red diamond) and 95% CI (blue horizontal line).

**Fig 5:**
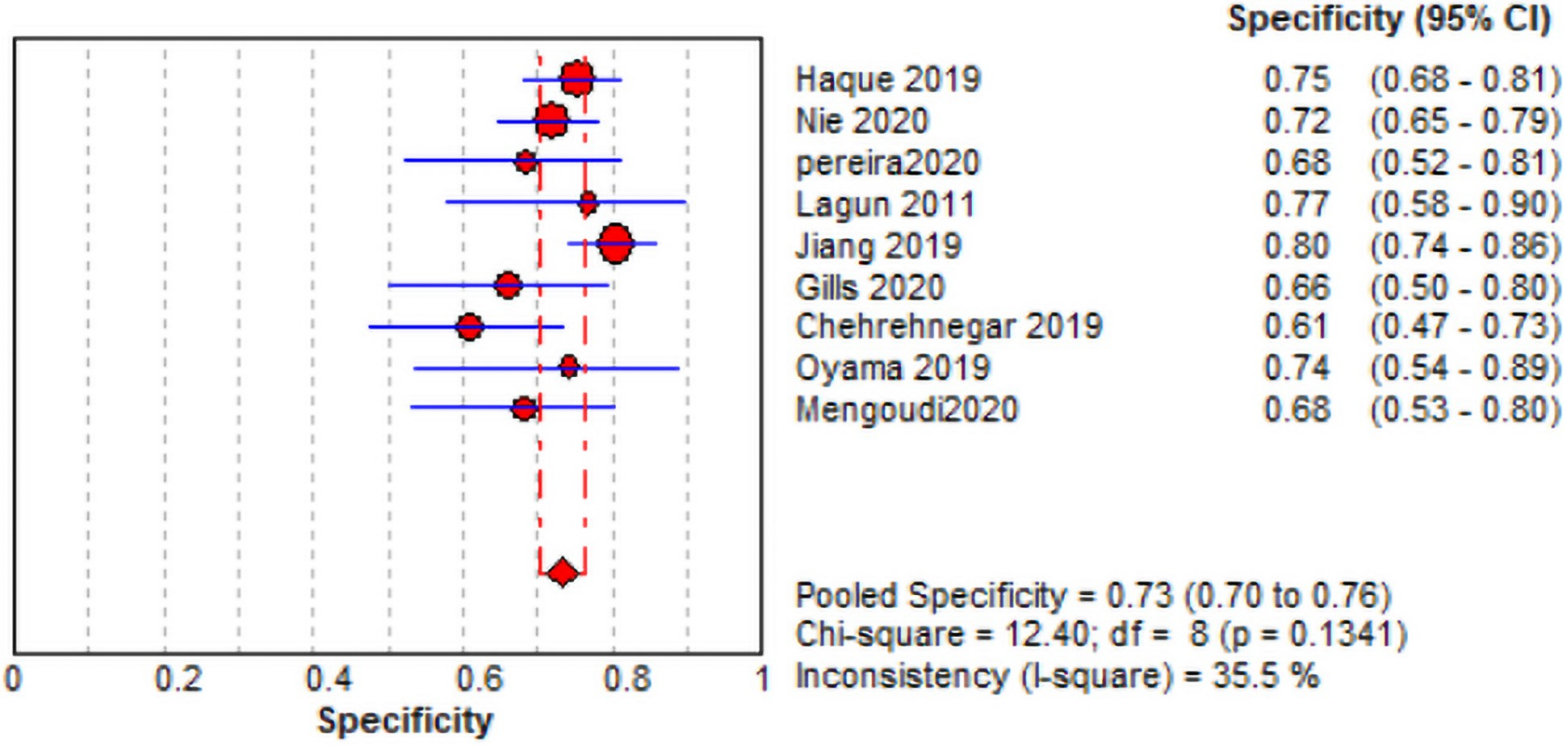
Specific (red diamond) forest map and 95% CI (blue horizontal line).

**Fig 6:**
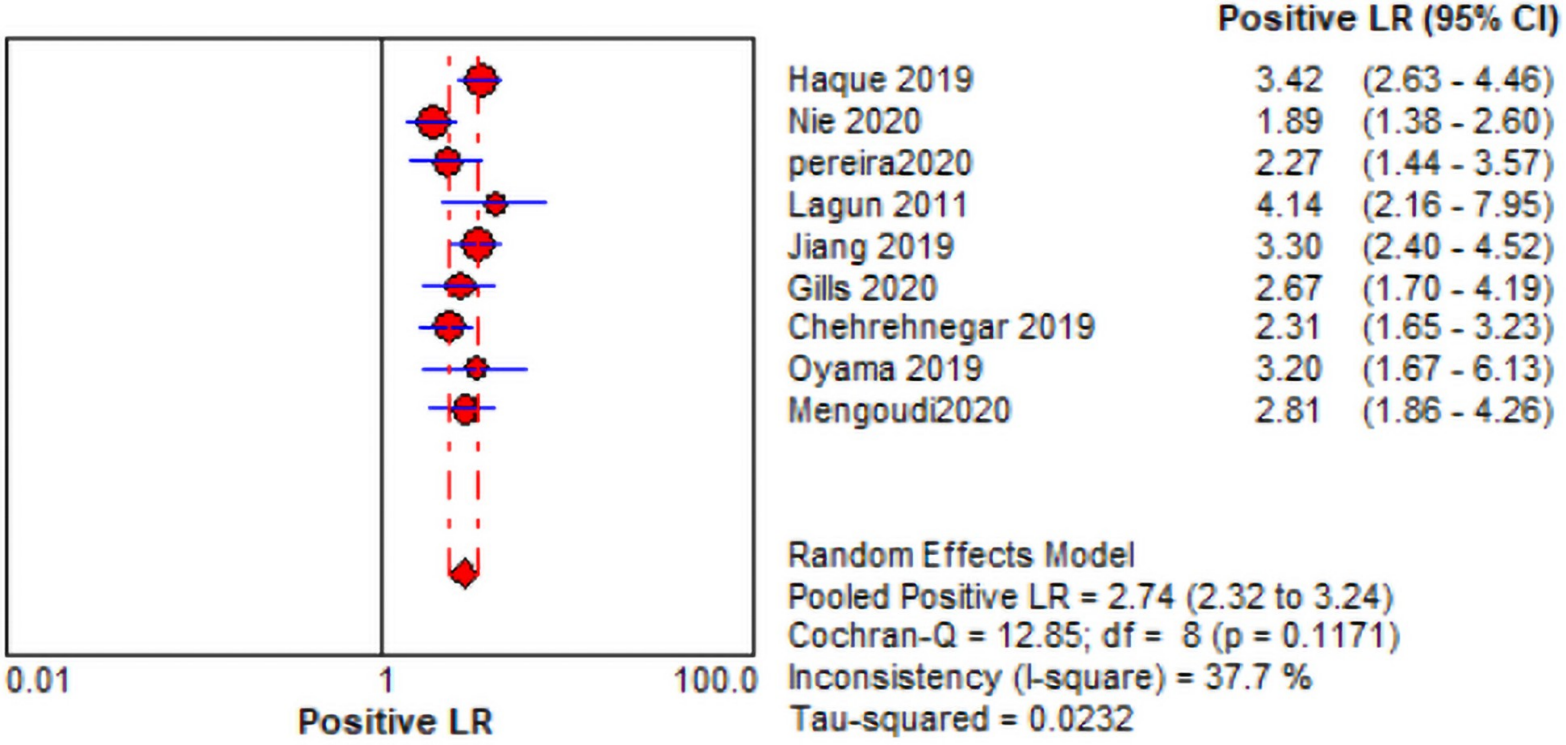
Forest map of LR+.

**Fig 7:**
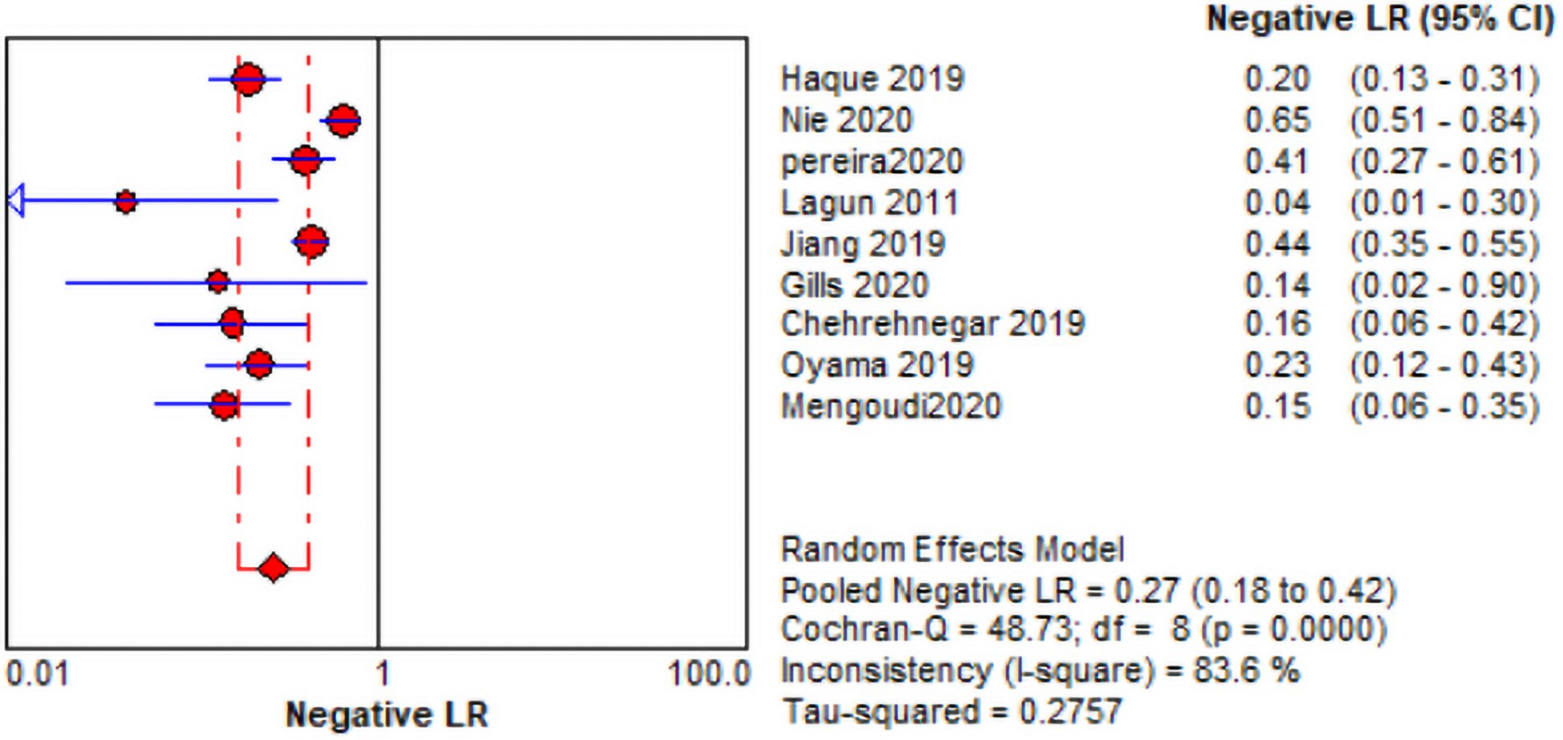
Forest map of LR-.

**Fig 8:**
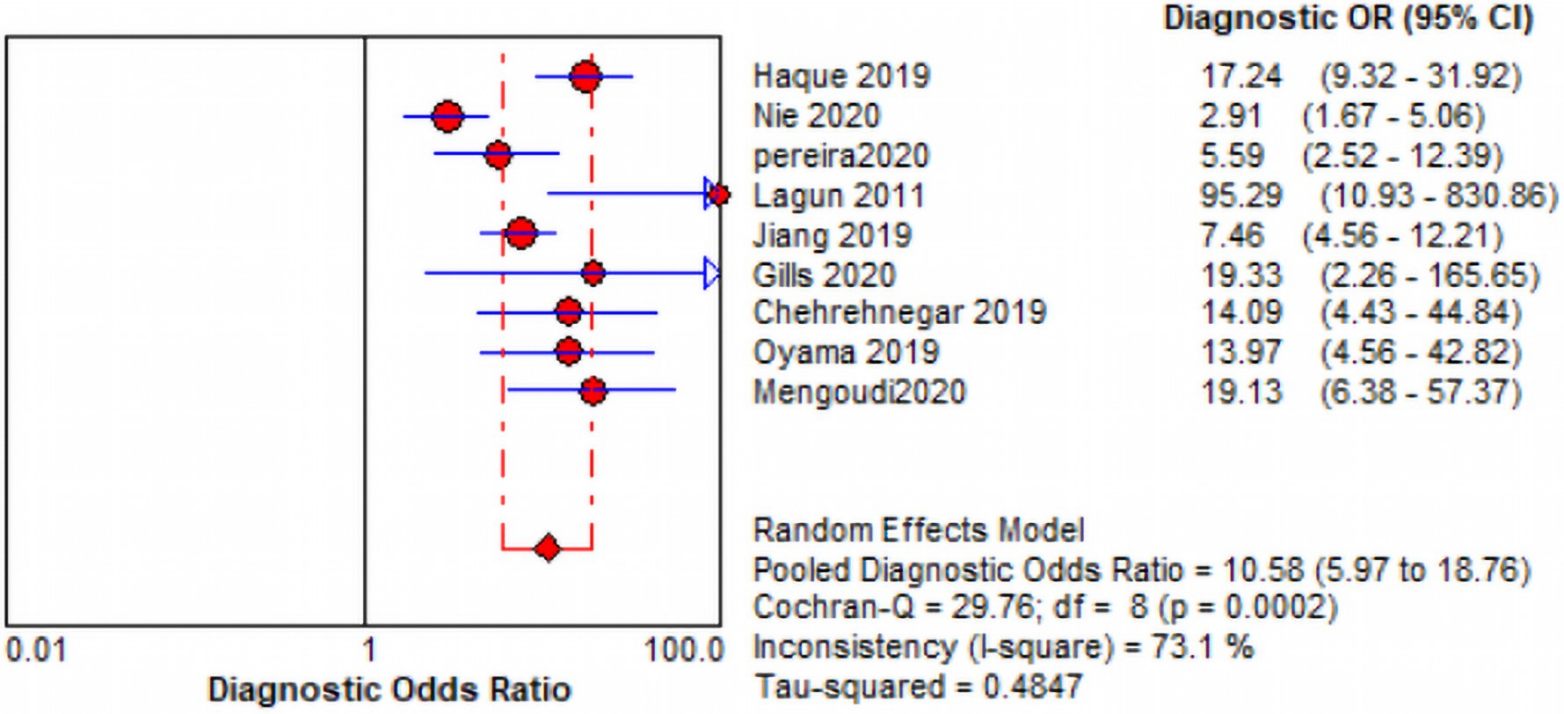
Forest map of DOR.

**Fig 9:**
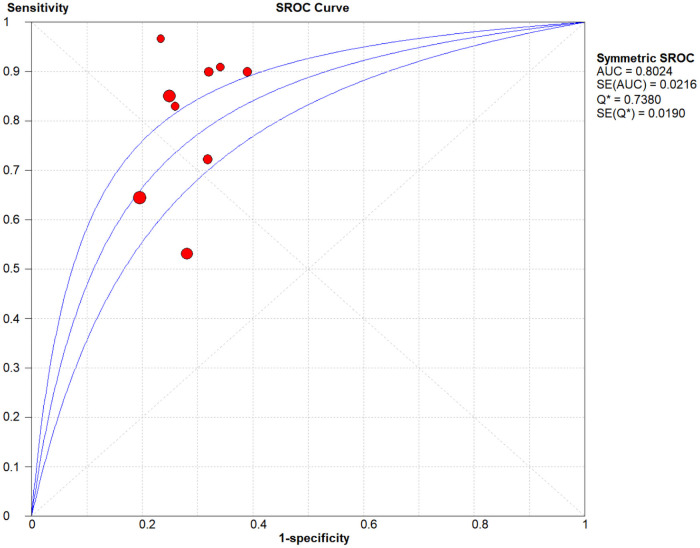
SROC with a 95% confidence interval.

**Fig 10:**
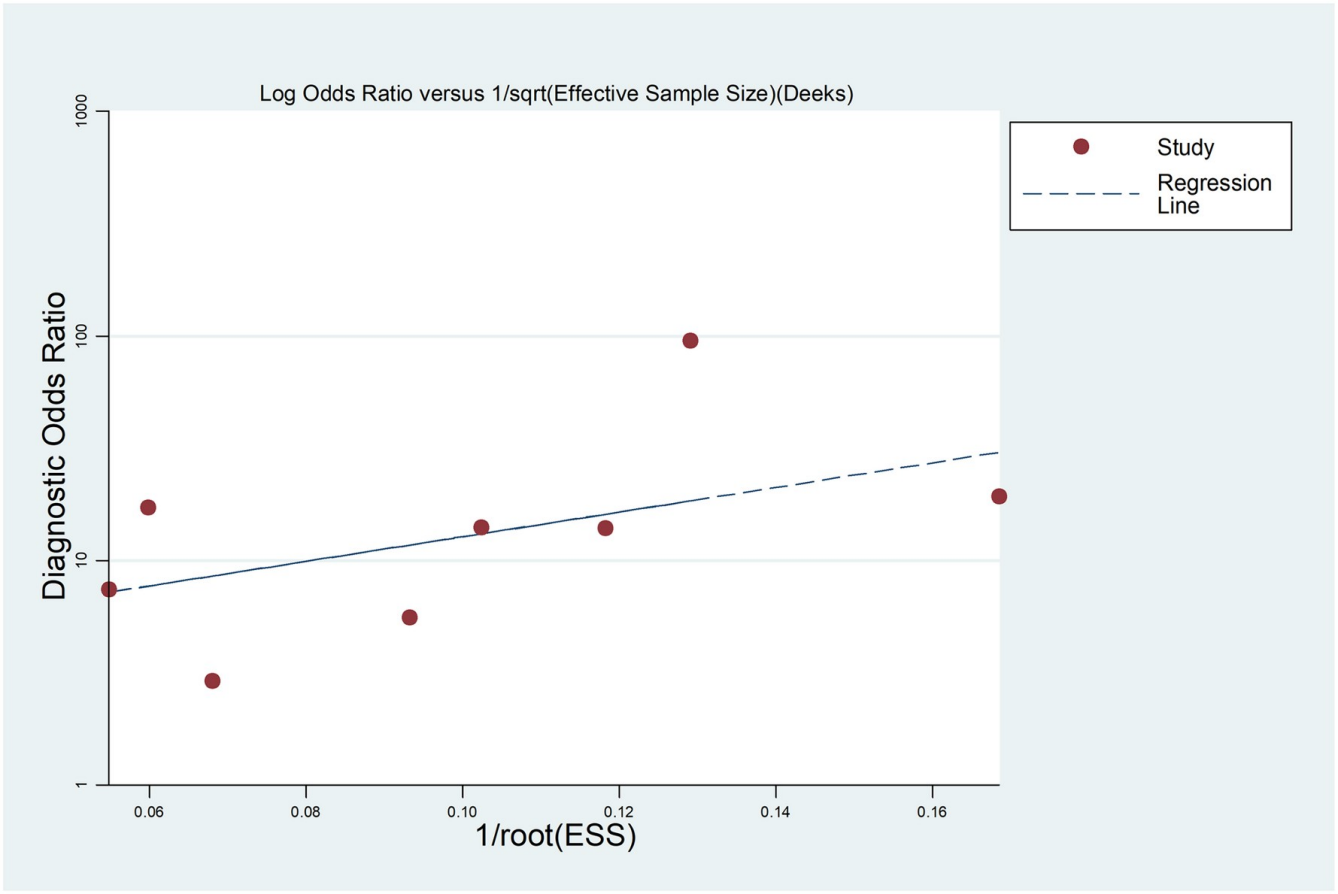
Deeks’ funnel plot for testing publication bias.

**Fig 11:**
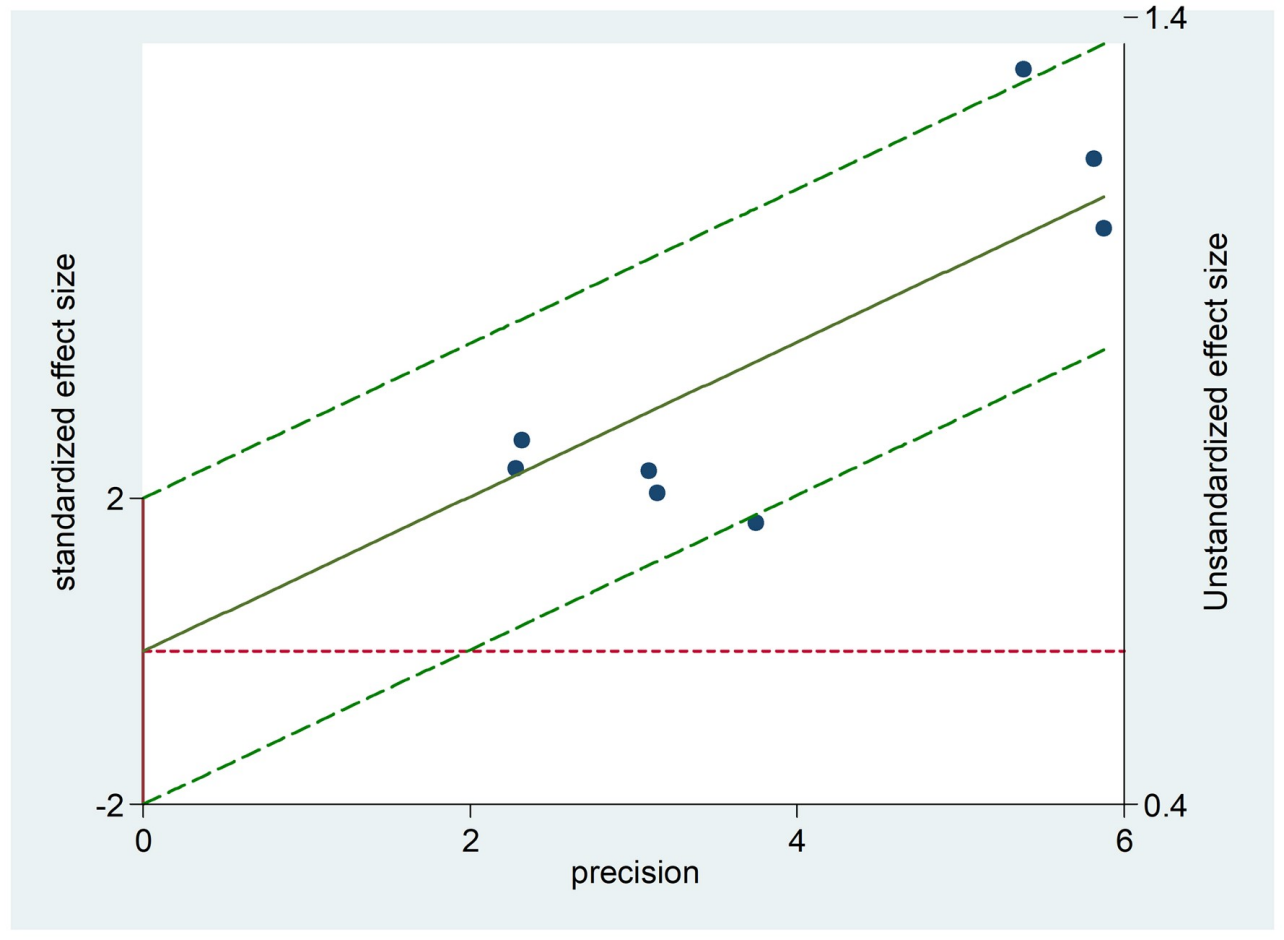
Galbraith diagram for heterogeneity analysis.

**Fig 12:**
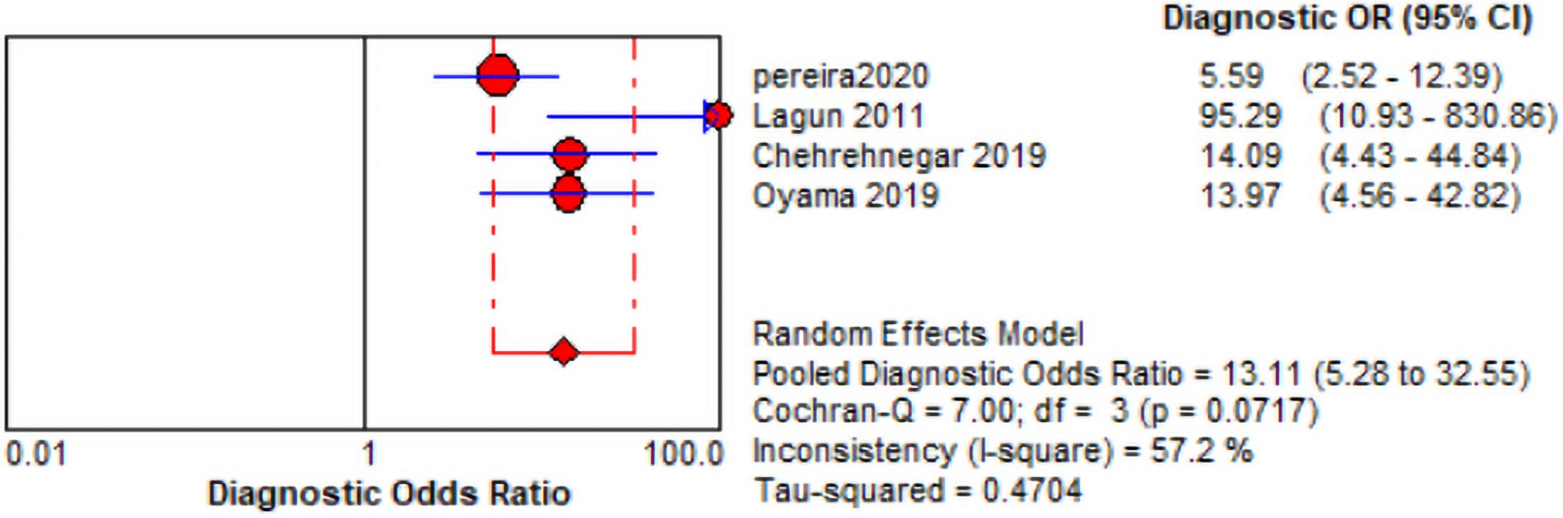
After subgroup analysis—Forest plot of the DOR.

The comprehensive forest plots of specificity, sensitivity, LR+, LR−, DOR, and SROC can be seen in Figs 4–9, respectively, and the total weighted area under the curve obtained by SROC analysis was 0.8024 (0.0216). the index Q-value was 0.7380 (0.0190), which is a strong indicator (0.7<ROC = 0.8024<0.9). The pooled sensitivity and specificity were 0.75 (95%CI 0.72–0.79) and 0.73 (95%CI 0.70–0.76; Figs 4 and 5), the pooled DOR of 10.58 (95% CI 5.97–18.76), the pooled LR+ was 2.74 (95% CI 2.32–3.24), and pooled LR− values was 0.27 (95% CI 0.18–0.42; Figs 6–8). The SROC curve in Fig 9 does not show a "shoulder and arm" pattern indicating that there was no threshold effect. Through Deek’s funnel plot (Fig 10) and Egger method (P-value = 0.291; Table 2), we inferred that there was no publication bias. We used the Galbraith diagram for heterogeneity analysis, 2 outliers were found (Fig 11). According to the results of the forest plot, there may be great heterogeneity. Subgroup analysis was performed on studies with epidemiology ≥0.5, and the results showed that the P-value of DOR was greater than 0.05 (P>0.05, Fig 12), indicating that epidemiology was one of the causes of heterogeneity, which may be connected with study design, sample size and the total number of the control group, etc. In addition, different eye movement tasks, parameters, and machine models may also contribute to the heterogeneity.

**Table 2:** Inferred by egger method that there is no publication bias(P-value = 0.291).

yb	Coef.	Std. Err.	t	P >l t l	95% conf. interval	
Bias	12.62263	10.90186	1.16	0.291	-14.05324	39.29851
Intercept	1.0283578	0.8879133	1.45	0.189	-0.8890679	3.456223

## Discussion

How can ET technology be useful for CI? That’s what we have to understand. Subjects when performing a certain task, eyes looking at the screen, the eye movement tracking can capture the eye looking at the screen when the various parameters, such as the amount of time when scanning the same pictures, rotational speed, distance, etc., and compared with the parameters of normal people, find out the differences, to judge whether the subjects have CI. Due to impaired visual space and executive functions and declining attention and memory, patients with cognitive impairment are different from normal people when observing static images, showing so different attention for different regions of the image that they cannot effectively explore each part of the image. These exploratory responses can reflect a person’s cognitive state. The change in the eye movement trajectory in patients with cognitive dysfunction is not a biological indicator rather than a state indicator, and therefore does not depend on the local subtle movements. As long as the patient displays retained the basic movement of the oculomotor nerve and muscles, ET remains a helpful tool [18]. Control of eye movement relies on extensive brain structures and networks which are often damaged during the disease [37, 38]. Eye-tracking metrics bridge brain behavioral function and neural mechanisms to reflect workings within the brain [39, 40]. For example, multiple areas of the cerebral cortex, superior colliculus, and thalamus can be activated when performing eye movement tasks [41, 42]. Eye movement disorders were considered to be effective in tracking the severity and progression of AD [43]. ET offers an objective means to assess motor cerebral involvement in ALS [44]. Impaired ET performance in patients with presenile onset dementia [45], and ET can measure disease progression in cognitively impaired patients [12]. With this background, we have conducted this systematic review.

For a doctor, recognizing CI at an early age has become an increasingly important challenge [46], our meta-analysis showed that ET technology could detect the decline in CI, which provides doctors with valuable information about patients’ CI, and early diagnosis is very important to them. The pooled sensitivity and specificity of ET for perceiving cognitive disorders were 0.75 and 0.73, respectively. Yet, we discovered that multiple elements required to be considered in the application of ET, and its part in cognitive disorders diagnosis should be interpreted. The causes of CI are complex, such as MCI, dementia, AD, and other neurodegenerative diseases. In fact, many studies have directly or indirectly demonstrated the effectiveness of ET technology in helping to diagnose, predict, or assess CI. However, meta-analysis was not performed because it failed to meet our criteria for inclusion in the quantitative analysis, but their results were equally important. These are some of the most authoritative and rigorous studies in the field that can’t be ignored. Clough et al. indicated that the task of working memory during eye movement can be used to distinguish multiple sclerosis (MS) patients with memory deficits from healthy individuals [47]. Cognitive assessment in patients with epilepsy requires a multifactorial and neurodevelopmental model because the oculomotor nerve test evaluates response inhibition and working memory through related tasks [39]. Some researchers believe that eye movement is a marker of CI in people with epilepsy [48]. Amador et al. study thinks ET tasks are associated with each other and the severity of the disease, suggesting that eye movement may be a useful tool in studying advanced cognitive functions [49]. Crawford et al. indicated that the decline of inhibitory control in the anti-saccade task (AST) might be a significant marker for interring working memory dysfunction in AD [50]. Archibald et al. study highlights the potential use of exploration strategy measures as a marker of cognitive decline in PD [51]. Chau et al. quantified novelty preference in AD patients by measuring visual scanning behaviors using an ET paradigm [52]. Ahonniska-Assa et al. assessed cognitive functioning in females with Rett syndrome by eye-tracking methodology [53]. MacInnes et al. established a cognitive state generation model based on tasks and eye movements [54]. Kaczorowska, M., et al. used eye-tracking to build cognitive models for the purpose of selecting the most significant feature, and the best result of these was 0.95 [55]. These results indicated the potential of eye movement tracking in the diagnosis of CI.

Although many diseases can lead to CI, the emphasis on CI varies from disease to disease. For example, some disorders are characterized by attention deficits, some are characterized by memory impairment, and some are characterized by executive function. Identifying the characteristics that affect cognitive function in a particular patient can help establish the cause of CI and the severity of the neurological disorders [56]. In addition, different studies have used different methods of ET. ET tasks consist of five basic types: namely, saccades, fixation, smooth pursuit, visual searching, and social cognition, among which, saccades and fixation are most commonly used. The saccade task also includes the front saccade task, the back saccade task, and the memory saccade task [49]. The parameters of these saccade tasks mainly reflect impairments in executive function [12]. In one study, the accuracy of a visual search task was also used to assess whether the executive function was impaired [44]. Girardi et al. Judgment of Preference based on eye gaze and recognition of Facial Expressions of Emotion to assess social cognition [57]. The VPC was considered by many studies to be a good method for memory recognition [26, 58, 59]. The cognitive impact of ET is increasing and significant progress has been made, despite the technical and methodological challenges of ET.

Overall, the quality of the studies we included was relatively modest (as identified by QUADAS-2). But the results and methods of these studies are reliable and rigorous. Our review has four major limitations. First, our analyses were performed based upon a few studies with distinct heterogeneity. Our results should be interpreted cautiously. Second, the methods in different studies of ET used in screening for CI are different, making it hard to reach clear conclusions nowadays. Third, restricting the search to publication in English may lead to the omission of some correlative literature. Finally, gray literature was not included in our review, which may lead to publication bias.

## Conclusions

In conclusion, our research indicated that eye-tracking technology could detect the decline in CI. The clinical use of eye-tracking technology in combination with other tools for the evaluation of cognitive disorders can be encouraged based on currently available evidence. This technology has not yet reached its maximum validity, and its methods, techniques, and the appropriate combination of parameters and indicators are still in the development stage. Available studies about the application of ET for CI diagnosis differ considerably, and the best protocol to implement ET in patients with cognitive disorders is being explored. More high-quality researches concerning ET examinations detecting CI are needed.

## Supporting information

S1 Checklist PRISMA 2009 checklist. (DOC)

S1 File Data availability and search strategy. (DOCX)
