# Supplementary material for: The effectiveness of eye tracking in the diagnosis of cognitive disorders: A systematic review and meta-analysis
Source: PLoS One. 2021 Jul 12;16(7):e0254059. doi: 10.1371/journal.pone.0254059 (PMC8274929; doi:10.1371/journal.pone.0254059)
Supplement: S1 File — (DOCX) [file pone.0254059.s002.docx]

**Pubmed/(MEDLINE)**

#1. “Cognitive Impairment” OR “cognitive functions” OR “cognition” OR “Cognitive dysfunction” OR “Cognitive decline” OR “cognitive disorders” 302353

#2. “eye-tracking” OR “gaze-tracking” OR “eye movement” OR “oculomotor” OR “fixation tracking” OR “saccade” OR “eye task” 56434

#3. "diagnose" OR "Diagnosis" OR "Sensitivity and Specificity" OR "diagnostic accuracy" OR "accuracy" OR "screening test" OR "sensitivity specificity" 4233046

#4. #1AND#2AND #3 707 results

**Embase**

1. “Cognitive Impairment” OR “cognitive functions” OR “cognition” OR “Cognitive dysfunction” OR “Cognitive decline” OR “cognitive disorders” 471260

2. “eye-tracking” OR “gaze-tracking” OR “eye movement” OR “oculomotor” OR “fixation tracking” OR “saccade” OR “eye task” 91486

3. "diagnose" OR "Diagnosis" OR "Sensitivity and Specificity" OR "diagnostic accuracy" OR "accuracy" OR "screening test" OR "sensitivity specificity" 6567840

#1. 1AND2AND 3 1722 results

**Cochrane**

Advanced Search

**Title Abstract Keyword:** “Cognitive Impairment” OR “cognitive functions” OR “cognition” OR “Cognitive dysfunction” OR “Cognitive decline” OR “cognitive disorders”

**AND Title Abstract Keyword**: “eye-tracking” OR “gaze-tracking” OR “eye movement” OR “oculomotor” OR “fixation tracking” OR “saccade” OR “eye task”

**AND Title Abstract Keyword:** "diagnose" OR "Diagnosis" OR "Sensitivity and Specificity" OR "diagnostic accuracy" OR "accuracy" OR "screening test" OR "sensitivity specificity"

**Run search**

**Trial 145** **results**

**Web of Science**

**# 1 283471** **results**

TS=(“Cognitive Impairment” OR “cognitive functions” OR “cognition” OR “Cognitive dysfunction” OR “Cognitive decline” OR “cognitive disorders”)

**# 2 55057** **results**

TS=(“eye-tracking” OR “gaze-tracking” OR “eye movement” OR “oculomotor” OR “fixation tracking” OR “saccade” OR “eye task”)

**# 3 2965446** **results**

TS=(“Cognitive Impairment” OR “cognitive functions” OR “cognition” OR “Cognitive dysfunction” OR “Cognitive decline” OR “cognitive disorders”)

**# 4 434** **results**

#3 AND #2 AND #1

**Data Availability：Review Manager 5.3 was used to calculate TN, FP, and FN according to the total sample size of sensitivity and specificity provided in the original paper.**
